# Using Eco-Friendly Recycled Powder from CDW to Prepare Strain Hardening Cementitious Composites (SHCC) and Properties Determination

**DOI:** 10.3390/ma13051143

**Published:** 2020-03-04

**Authors:** Wan Wang, Huixia Wu, Zhiming Ma, Ruixue Wu

**Affiliations:** 1 Department of Civil Engineering, Tongji Zhejiang College, Jiaxing 314051, China; wangwan@tongji.edu.cn; 2 College of civil science and engineering, Yangzhou University. Yangzhou 225127, China; dyyang_sub@163.com (H.W.); Ruixue_WuQT@163.com (R.W.); 3 Department of Structural Engineering, Tongji University, Shanghai 200092, China

**Keywords:** strain hardening cementitious composites (SHCC), construction and demolition waste (CDW), recycled brick powder (RBP), properties determination

## Abstract

Using eco-friendly recycled brick powder (RBP) derived from waste brick to prepare strain hardening cementitious composites (SHCC) provides a new way of recycling the construction and demolition waste (CDW), and the dosage of cement in SHCC can be decreased. This paper investigated the micro-properties and mechanical properties of SHCC containing RBP by a series of experiments. The results showed that RBP had typical characteristics of supplementary cementitious material (SCM). The addition of RBP increased the SiO_2_ content and decreased the hydration products in cementitious materials; in this case, the mechanical properties of mortar decreased with increasing RBP replacements, and a linear relationship was observed between them. It was noticed that the adverse effect of RBP on the mechanical properties decreased with increasing PVA fiber content in mortar. For SHCC containing various RBP replacements, the ultimate load increased, and the ultimate displacement decreased with increasing curing days. When using RBP to replace cement by weight, the ultimate displacement increased with the addition of RBP. Meanwhile, there was no significant reduction in the ultimate load of SHCC. When using RBP to replace fly ash (FA) by weight, the incorporation of RBP decreased the ultimate displacement of SHCC, whereas the ultimate load was improved. For example, the ultimate load and displacement of SHCC with 54%RBP were 17.6% higher and 16.4% lower, respectively, than those of SHCC with 54% FA.

## 1. Introduction

The rapid onset of global urbanization has resulted in a massive number of construction and demolition wastes (CDW) [1]. In China, the output of CDW reached approximately 1.8 billion tons per year, and it will be kept at a high level in the next few years [2]. Because of CDW containing some hazardous substances, the traditional disposal of CDW by dumping has inevitably resulted in environmental pollution, such as soil and water pollution [3,4]. Therefore, disposing such CDW in effective and eco-friendly ways has become an important issue around the world [5]. In recent years, recycling technologies for CDW are developed, and the waste concrete and brick are recycled into recycled aggregate and used to prepare recycled aggregate concrete [6,7]. The properties of recycled aggregate from waste concrete are similar to those of natural aggregate, the recycled aggregate has been widely used in the construction engineering, and the high-quality recycled aggregate can also be employed in the preparation of high performance recycled concrete [8,9], self-compacting recycled concrete [10], and structural recycled concrete [11]. Moreover, the improvement technology for the recycled concrete aggregate and prepared concrete has also developed in recent years, whereby excellent properties can be obtained after enhancement treatment [12,13]. Being different from the recycled concrete aggregate, the utilization of recycled brick aggregate in construction engineering is very scarce because of recycled brick aggregate containing high porosity and water absorption; in this case, the recycling rate of waste brick was much lower than that of waste concrete in CDW [14]. 

Forcing on the challenge of recycling the waste brick in CDW, some scholars proposed using waste bricks to prepare recycled brick powder that can be used as supplementary cementitious material (SCM) in the preparation of cement and concrete products, which was a sustainable cement-based material [15,16]. Some investigations have been conducted to study the properties of RBP and prepared concrete. For example, findings have been reported that the main compositions of RBP are similar to those of fly ash; attributed to RBP containing high content of amorphous phase and vitreous phase, the high fineness RBP has a satisfied pozzolanic activity [17,18]. Incorporating an appropriate content of high activity RBP has less impact on the mechanical properties of prepared concrete, and even a slight increase in the mechanical properties can be observed with the addition of high fineness RBP. However, the incorporation of high-volume RBP significantly decreases the mechanical properties [19] For example, Olofinnade et al. [20] found that the compressive strength and tensile strength of concrete with 10% RBP were respectively 32% and 2% higher than those of the plain concrete after 90 d curing. In addition, the RBP concrete has a better durability performance than the plain concrete, as Ma et al. [21] and Ge et al. [22,23] found that the RBP concrete has better water penetration and sulfate attack resistance than the plain concrete. Therefore, using RBP as SCM to prepare concrete is appropriate, and the RBP is sustainably recycled material.

To address the brittleness issue on the ordinary concrete materials, the strain hardening cementitious composites (SHCC) have been developed due to the fiber-bridging action of multiple cracking behaviors and ultra-high ductility [24,25]. Investigations reported that the SHCC possessed excellent mechanical properties and durability performance [26,27,28]. However, it is noticed that more than half of SHCC component, according to the mix proportion by weight, is the cement and the SCM of fly ash (FA) or silica fume (SF) [29,30]; in this case, the preparation cost is increased, and SHCC is not eco-friendly due to the addition of massive cement. Thus, an eco-friendly and sustainable SHCC is urged to be developed. Considering that the RBP could be used as the SCM, one can expect that the RBP could be further used in the preparation of SHCC. The utilization of RBP in SHCC not only decreases the dosage of cement and FA in SHCC, but also promotes the recycling rate of the waste brick in CDW, which is eco-friendlier and more sustainable.

Based on the introductions above, this paper was developed to investigate the properties of SHCC containing RBP. The RBP was respectively used to replace the cement and FA in SHCC by weight, and the micro-properties of cementitious materials and the mechanical properties of SHCC with RBP were determined. The authors hope the findings in this paper are helpful to further research on the SHCC and CDW recycling.

## 2. Materials and Experimental Details

### 2.1. Preparation of the RBP Derived from the CDW

**Figure 1:**
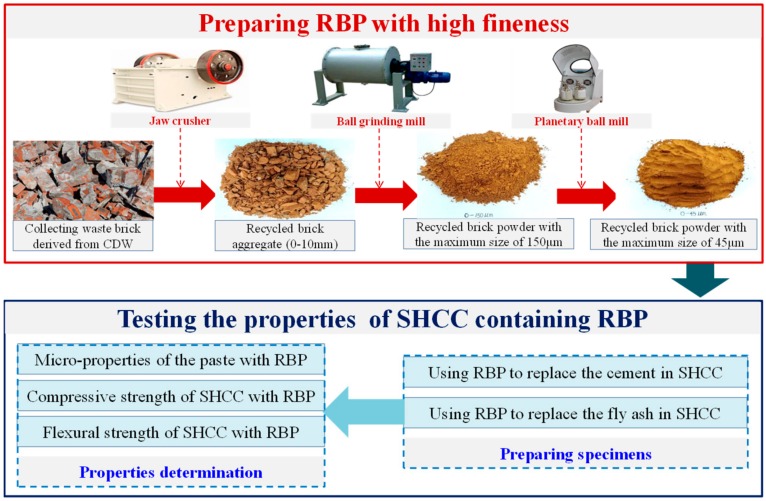
Flow diagram of using the waste bricks in CDW to prepare RBP.

Figure 1 shows the flow of using waste bricks to prepare high fineness RBP. The waste bricks were first collected from the CDW by machine or manual sorting, and then, they were crushed into the recycled brick aggregates with a maximum size of 10 mm by a jaw crusher (SANME, Shanghai, China). Subsequently, the recycled brick aggregates were ground to low fineness RBP with a maximum size of 150 μm by a ball grinding mill (SANME, Shanghai, China), and the obtained RBP were further ground to high fineness RBP with a maximum size of 45 μm by a planetary ball mill (Jingxin, Shanghai, China). The high fineness RBP was used in the preparation of SHCC. Furthermore, Figure 1 shows the test flow in this paper. The effects of fiber content and RBP replacements on the properties of cementitious materials were considered in the experiment design. The micro properties of cementitious materials containing RBP were determined to reveal the feasibility of using RBP as SCM in the SHCC preparation, and the compressive and flexural strength were tested to investigate the mechanical properties of SHCC with RBP.

### 2.2. Mix Proportion and Specimens Preparation

Table 1 gives the mix proportion of the mortar and SHCC with RBP. The mortar specimens were prepared, to quantify the effects of RBP and fiber on the properties of cementitious materials. The mix proportion of SHCC with RBP was proposed according to that of SHCC with FA by the previous studies; the total content of binding materials was 1200 g, and the content of water, sand and PVA fiber is respectively 395 g, 550 g, and 26 g [31,32,33,34]. On the one side, the RBP was used to replace the cement in SHCC by weight, and the replacement ratios (weight) were 0% (0 g), 18% (216 g), 36% (432 g), and 54% (650 g), which aimed to investigate the effects of RBP content on the properties of SHCC, such as, the sample titled SHCC-54%RBP represents that 54% of cement in SHCC was replaced by the same weight of RBP. On the other side, the RBP was used to replace the FA by weight, which aimed to investigate the potential of using RBP to replace the FA in the preparation of SHCC; for example, the sample SHCC-27%RBP + 27%FA represented that the RBP and FA replacements are both 27%. 

It is noticed that the mix proportion of the mortar is corresponding to the mix proportion of SHCC in this paper, and the mortar is also titled as the SHCC without PVA fiber; in this case, the effect of PVA fiber content on the mechanical properties of SHCC can be well quantified. Besides, the pozzolanic activity of RBP is also determined according to British standard BS EN 450-1 and Chinese standard GB/T 1596-2017, to prove the feasibility of using RBP as SCM in SHCC. The amount of cement, RBP, water and sand in this mix proportion is respectively 315, 135, 225 and 1350 kg/m^3^, and the pozzolanic activity index of RBP is determined as the value of 89.6% by a compressive strength test. Therefore, the high fineness RBP used in this study has high pozzolanic activity. 

Referring to the related references on the determination of SHCC properties, the sample size was 40 × 40 × 160 mm, and a three-point bending test was conducted to determine the flexural strength and ductility of SHCC with RBP [35,36]. All the specimens were first prepared according to the mix proportion shown in Table 1, and the specimens after 24 h hardening were placed in the standard curing room (T = 20 ± 2 °C, RH ≥ 95%) for 3 d, 14 d, and 28 d. When reaching the targeted curing days, the mechanical properties of the mortar and SHCC samples were determined. 

### 2.3. Micro Properties and Mechanical Properties Determination

The paste with RBP was used in testing the micro properties of cementitious materials. The test of SEM (scanning electron microscope, Hitachi, Tokyo, Japan) was applied to show the micro-structure of cementitious materials, and the elemental map was further presented to quantify the elemental composition of cementitious materials; moreover, the XRD (X-ray diffraction, Bruker AXS, karlsruhe, Germany) test and TG (thermogravimetric, PerkinElmer, Waltham, MA, USA) test were conducted to reveal the effect of RBP incorporation on the hydration products of cementitious materials [37,38]. The mortar and SHCC samples were used to test the mechanical properties, including the compressive strength and flexural strength, and the mechanical test was conducted according to Chinese standard GB50081 (2019) and American standard ASTM-C78 (2005). The applied load in the bending test was controlled by the displacement, and the loading rate was kept at 0.05 mm/min until the sample failure. The applied force and displacement were simultaneously obtained by the testing machine (MTS, Eden Prairie, MN, USA); and in this case, the force-displacement curves can be obtained, and the ultimate force and ultimate displacement were used in this paper to evaluate the mechanical properties of SHCC containing RBP. 

## 3. Results and Discussion

### 3.1. Micro-Properties of the Cementitious Materials Containing RBP

**Figure 2:**
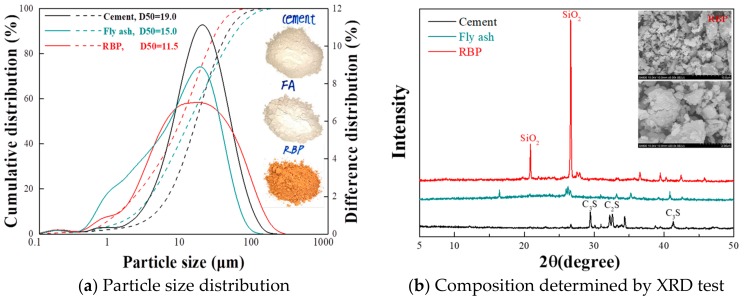
Properties of CBP used in the preparation of SHCC. (**a**) Particle size distribution; (**b**) Composition determined by XRD test.

This section aims to investigate the feasibility of using RBP as SEM in SHCC preparation. The high fineness powder frequently results in a high activity, and the high fineness powder is helpful to promote the micro-aggregate filling effect of cementitious materials [39]. Considering that the RBP is derived from the waste brick and no hydrated substance contained in it, the fineness of RBP should be higher than that of the cement and FA, which is beneficial to improve the activity of RBP when using RBP as SCM to replace the cement or FA in SHCC. Figure 2a shows the particle size distribution of cement, FA and RBP used in this paper, and the results show that their median diameter (D50) is approximately 19 μm, 15 μm, and 11.5 μm. Figure 2b shows the XRD results, whereby the SiO_2_ content in RBP is much higher than that in cement and FA, and there are no hydrated compounds (such as CaO, Ca(OH)_2_) in RBP. Attributed the SiO_2_ being the main component that takes part in the pozzolanic reaction of cementitious materials, the high fineness RBP can be used as the SCM in the cementitious materials [40]. Figure 2b further shows the SEM images of RBP, and an irregular micro-structure can be observed.

**Figure 3:**
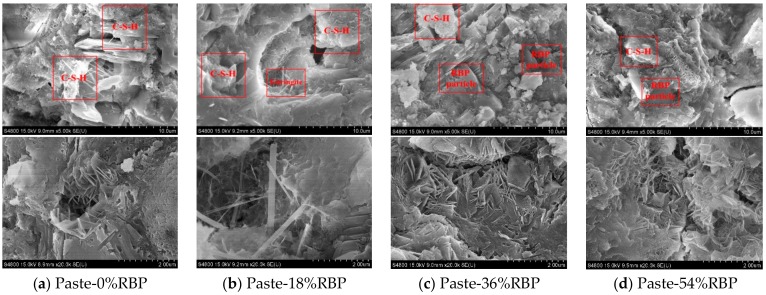
SEM images of paste with various replacements of RBP. (**a**) Paste-0%RBP; (**b**) Paste-18%RBP; (**c**) Paste-36%RBP; (**d**) Paste-54%RBP.

Figure 3 shows the micro-structure of the paste with RBP. Most C-S-H can be observed when the RBP replacement is 0%, whereas the content of C–S–H and Ca(OH)_2_ decrease with increasing RBP replacement. The content of ettringite and RBP micro-particle increases with the increase of RBP content, and the RBP micro-particle connects with the C–S–H in the paste. Attributed to the dilution effect and nucleation of RBP, the addition of RBP promotes the hydration reaction rate of cementitious materials. However, the RBP has higher SiO_2_ content and lower CaO content than the cement, and thus, incorporating RBP leads to a reduction in the hydration products when using RBP to partially replace the cement in the paste, and a similar phenomenon is obtained by previous studies [41,42]. When the RBP replacement is 18%, there is no obvious difference for the micro-structure of Paste-0%RBP and Paste-18%RBP, and this may be because the micro-aggregate filling effect of RBP filled the pores and meanwhile the reduction of hydration products in cementitious materials is not obvious. However, with a significant reduction of the hydration products in cementitious materials, the micro-structure of paste-54%RBP is looser than that of the paste-0%RBP, which indicates that the addition of high-volume RBP is adverse to the properties of prepared mortar and concrete.

**Figure 4:**
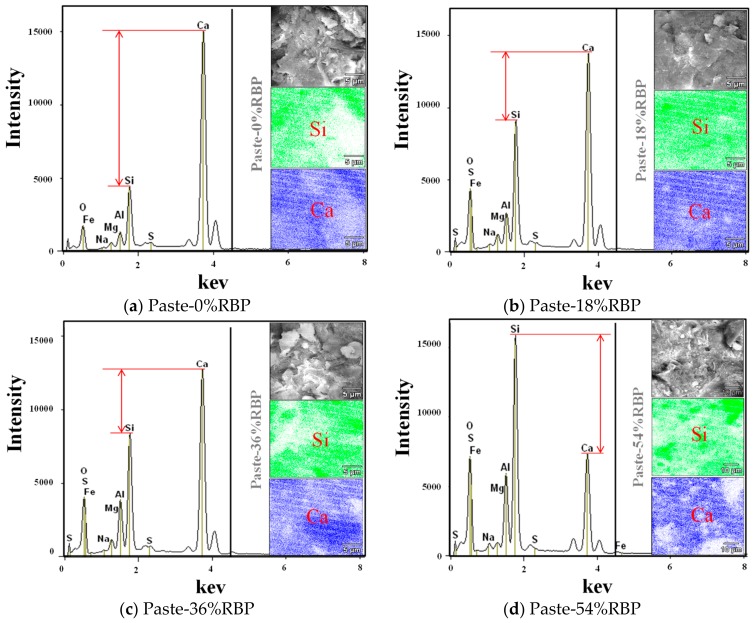
Elemental maps of the paste with various RBP replacements. (**a**) Paste-0%RBP; (**b**) Paste-18%RBP; (**c**) Paste-36%RBP; (**d**) Paste-54%RBP.

Figure 4 shows the elemental maps of the paste with various RPB replacements. When the RBP replacement ratios are below 36%, the calcium content is higher than the silicon, and the calcium content decreases, and the silicon content increases with the increase of RBP replacement ratios. In particular, the calcium content is lower than the silicon when the RBP replacement ratios are 54%. This may be because the RBP contains higher silicon content and lower calcium content than the cement, the silicon content increases, and the calcium content decreases with the addition of RBP in the paste. The appropriate calcium-silicate ratio is beneficial to the properties of cementitious materials, whereas the excessively low of calcium-silicate ratio results in the reduction of hydration products and is adverse to the properties of cementitous materials [43,44].

**Figure 5:**
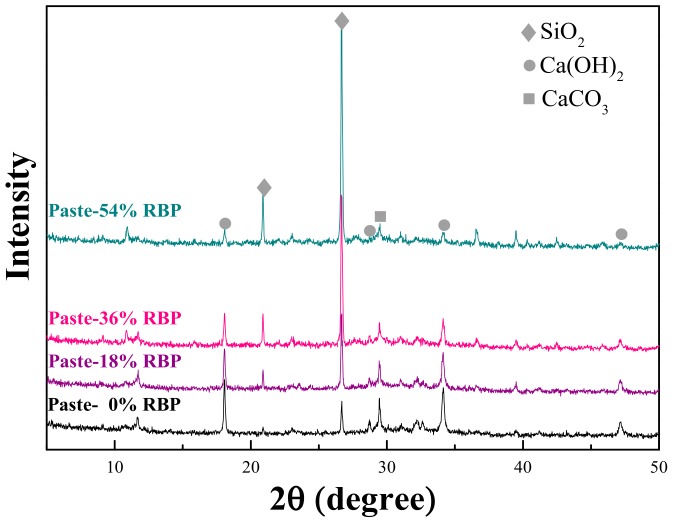
Compound composition of paste with various replacement of RBP by XRD test.

Figure 5 shows the XRD results of the paste containing RBP. The incorporation of RP increases the content of SiO_2_ in the paste, while the content of Ca(OH)_2_ and CaCO_3_ decreases with the addition of RBP. The Ca(OH)_2_ and CaCO_3_ are the main hydration products of cementitious materials; thus, incorporating RBP reduces the hydration products of cementitious materials [41,45]. Attributed to RBP containing higher SiO_2_ content and lower CaO content compared with the cement, the addition of RBP increases the SiO_2_ content when using the RBP to replace the cement by weight in the paste. The micro-particle SiO_2_ is the main compound that takes part in the pozzolanic reaction; thus, incorporating RBP promotes the pozzolanic reaction of cementitious materials, and the high fineness possesses an excellent micro-aggregate filling effect. However, the incorporation of RBP reduced the hydration products in the paste, which is adverse to the properties of cementitious materials. Therefore, the positive and negative effects of RBP on the micro-properties should be both considered in the performance analysis of SHCC.

**Figure 6:**
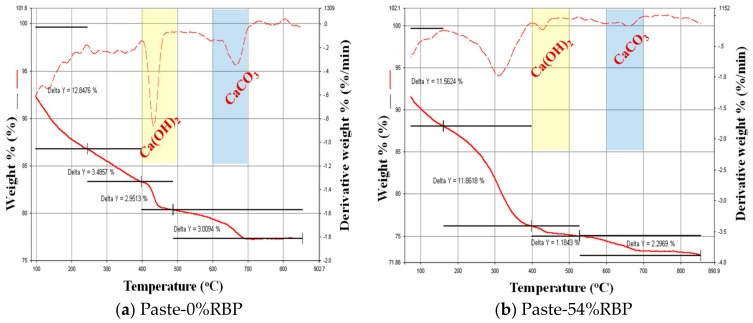
Hydration product analysis for the paste with RBP by DTG test. (**a**) Paste-0%RBP; (**b**) Paste-54%RBP.

Figure 6 shows the DTG (derivative thermogravimetric analysis) results of the paste with RBP after 28 d curing. Three peaks around 100 °C, 450 °C and 700 °C can be seen from the DTG curves. The first peak presents the dehydration of C–S–H gel, and incorporating RBP reduces the content of C–S–H gel in paste, which further proved that the incorporation of RBP decreases the hydration reaction of cementitious materials. The content of Ca(OH)_2_ and CaCO_3_ in the Paste-54%RBP is much lower than that in the Paste-0%, and a similar conclusion can also be obtained from Figure 5. As shown in Figure 2, Figure 3, Figure 4 and Figure 5, the micro-properties results of the RBP and prepared paste highlight that the high fineness RBP can be used as the SCM in the preparation of cement products. One can expect that the RBP can also be used in the preparation of SHCC, and the mechanical properties of SHCC containing RBP are discussed in the following sections.

### 3.2. Compressive Strength of the Mortar and SHCC Containing RBP

**Figure 7:**
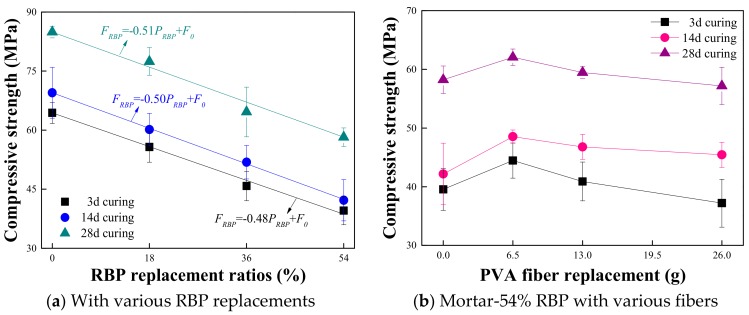
Compressive strength of mortar with vaious contents of RBP and PVA fiber. (Displayed values are the mean values of three measurements). (**a**) With various RBP replacements; (**b**) Mortar-54% RBP with various fibers.

The effects of RBP and PVA fiber content on the compressive strength of prepared mortar are first determined, and the results are shown in Figure 7. The compressive strength of the mortar with and without RBP both increased with increasing curing days. However, after undergoing the same curing days, the addition of RBP decreases the compressive strength; such as, the compressive strength of the mortar with 18% RBP, 36% RBP, and 54% RBP is 8.8%, 23.9%, and 31.4% lower respectively, than that of the control group without RBP. This may be because the incorporation of RBP decreases the hydration products in mortar and then the compressive strength is decreased. Besides, a linear relationship can be observed between the RBP replacement ratios and compressive strength of prepared mortar and the specific equation is described in Figure 7a, where the *F_RBP_* and *F_0_* represent the compressive strength of the mortar with and without RBP, in MPa; P_RBP_ is the RBP replacement ratios, in %. 

Figure 7b shows the compressive strength of the Mortar-54%RBP with various PVA fiber contents. It can be seen that the compressive strength of Mortar-54%RBP first increases and then decreases with increasing PVA fiber content, and the Mortar-54%RBP with 6.5 g PVA fiber has the highest compressive strength compared with that with the other contents of PVA fiber; such as, the compressive strength of Mortar-54%RBP with 6.5 g, 13 g, and 26 g PVA fiber is 6.6% higher, 2.1% higher, and 1.8% lower, respectively, than that without PVA fiber. This may be because the appropriate content of PVA fiber improves the integrality of prepared mortar and thus the compressive strength is increased; however, the high content of PVA fiber results in an obvious weak interface between the fiber and the paste, which is adverse to the compressive strength of prepared mortar [46].

**Figure 8:**
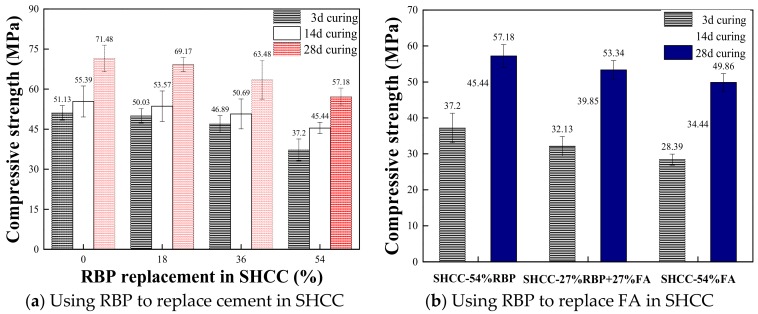
Compressive strength of SHCC with various RBP replacements. (Displayed values are the mean values of three measurements). (**a**) Using RBP to replace cement in SHCC; (**b**) Using RBP to replace FA in SHCC.

Figure 8a shows the compressive strength of SHCC when using RBP to replace cement by weight. The results show that the compressive strength of SHCC decreases with the increase of RBP replacements after undergoing the same curing days; such as, the compressive strength of SHCC-18%RBP, SHCC-36%RBP, and SHCC-54%RBP is 3.2%, 11.2%, and 20.0% lower, respectively, than that of the control group without RBP after 28d curing. Compared with the results in Figure 7a and Figure 8a, the adverse effect of RBP replacement on the SHCC strength is lower than that on the mortar strength; such as, the compressive strength of Mortar-54%RBP and SHCC-54%RBP is 23.9% and 20.0% lower than the control group after 28 d curing. Figure 8b further shows the compressive strength of SHCC when using RBP to replace the FA by weight. The addition of RBP increases the compressive strength of the SHCC with FA; such as, the compressive strength of SHCC-54%RBP and SHCC-27%RBP + 27%FA is 14.7% and 7.0% higher than that of SHCC-54%FA. This may be due to that RBP contained higher content of micro-particle SiO_2_ than the FA, and the incorporation of SiO_2_ with high micro-hardness is helpful to the strength of prepared SHCC. Therefore, when the replacement ratios of RBP and FA are the same, the compressive strength of SHCC with RBP is better than that of SHCC with FA.

### 3.3. Flexural Strength of SHCC When Using RBP to Replace Cement by Weight

**Figure 9:**
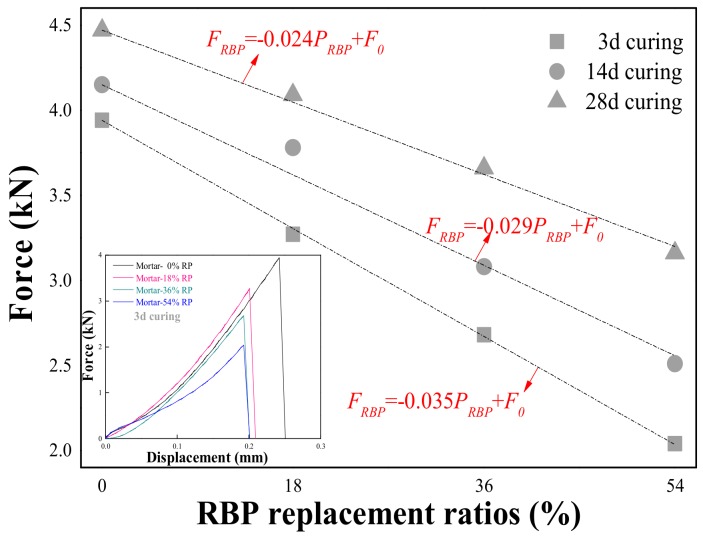
Flexural strength of mortar containing RBP after various curing days. (Displayed values are the mean values of three measurements).

The flexural strength of the mortar with various RBP contents was first determined, and the results are shown in Figure 9. The ultimate load of the mortar decreases with the increase of RBP replacements; such as, the ultimate load of Mortar-18%RBP, Mortar-36%RBP, and Mortar-54%RBP is 8.5%, 18.1%, and 29.3% lower, respectively, than that of Mortar-0%RBP when the curing duration is 28 d. In particular, a linear relationship can be observed between the RBP replacement and ultimate load of prepared mortar, and the detailed equation is also described in this figure, where the *F_RBP_* and *F_0_* represent the ultimate load of the mortar with and without RBP, in kN; the *P_RBP_* is the RBP content, in %. Figure 9 further shows the force-displacement curves of the mortar with various RBP replacements, and a similar ascent stage can be observed when the RBP replacements are below 36%; however, the ascent stage of Mortar-54%RBP was lower than that of the other samples, and possibly because the significant reduction of hydration products in mortar and the cementation is obviously decreased.

**Figure 10:**
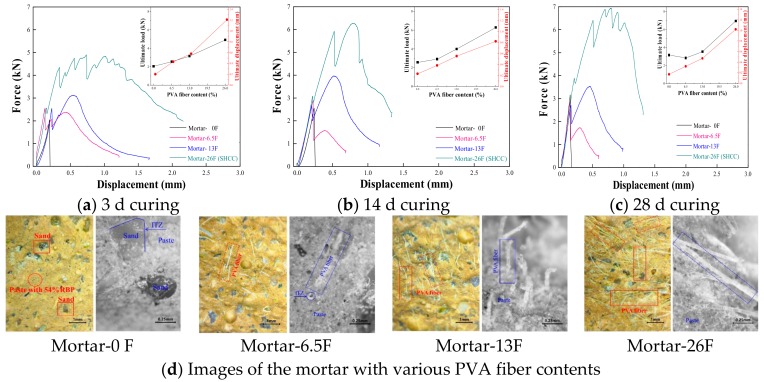
Force-displacement curves of mortar with various content of PVA fiber. (**a**) 3d curing; (**b**) 14 d curing; (**c**) 28 d curing; (**d**) Images of the mortar with various PVA fiber contents.

Figure 10 shows the force-displacement curves of the mortar with various PVA fiber contents under the application of flexural load. The results highlight that the ultimate load and displacement increase with increasing PVA fiber content in mortar; such as, after 3 d curing, the ultimate load of Mortar-6.5F, Mortar-13F, and Mortar-26F is 24.8%, 53.7%, and 140.3% higher, respectively than that of Mortar-0F, and the results are 115.0%, 190.0%, and 505.0% for the ultimate displacement. This is because the bridging effect provided by the PVA fiber in cementitious materials, and the ultimate strength and strain are improved [47]. Compared with the results in Figure 10a–c, the increasing curing days improve the ultimate load of the mortar with RBP, whereas the ultimate displacement decreases with increasing curing days. In Figure 10a–c, for the plain mortar without RBP, a strain-softening curve can be observed, and there is only one peak in the force-displacement curve. For the mortar with 6 g and 13 g of PVA fiber, two peaks can be observed from the force-displacement curve, and the first peak is the highest point for the Mortar-6F, however, the second peak was the highest point for the Mortar-13F. For the mortar with 26 g of PVA fiber as well as titled SHCC sample, an obvious strain hardening curves can be observed from the force-displacement curve of Mortar-26F. Because of the existence of high content of PVA fiber in cementitious materials, the applied flexural load can be well dispersed by the bridging stress between the PVA fiber and cementitious materials, more micro cracks, rather than the large crack, are produced in cementitious materials, and thus the strain hardening characteristic appears [48,49,50]. 

**Figure 11:**
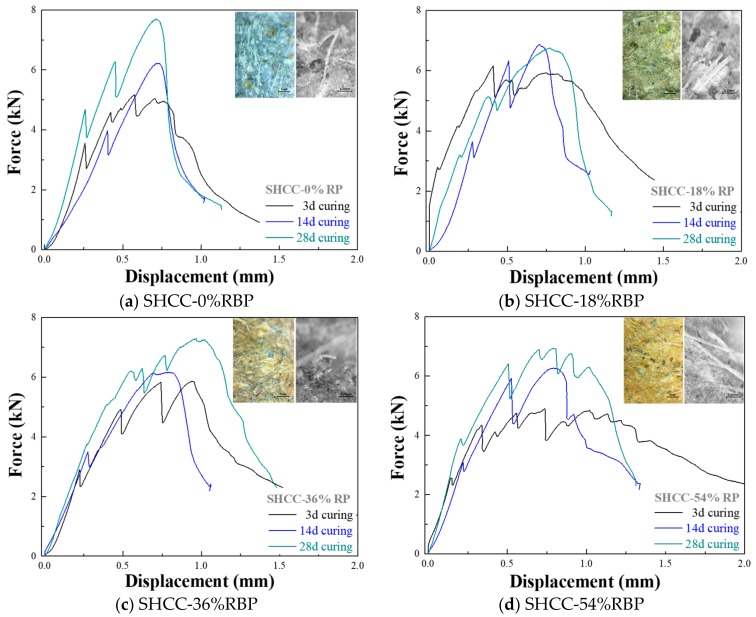
Force-displacement curves of SHCC containing RBP after various curing days. (**a**) SHCC-0%RBP; (**b**) SHCC-18%RBP; (**c**) SHCC-36%RBP; (**d**) SHCC-54%RBP.

To investigate the effect of curing duration on the flexural performance of SHCC with RBP, Figure 11 shows the force-displacement curves of SHCC with RBP after various curing days. The results indicate that the ultimate load increases with increasing curing days; for example, when the curing duration is after 3 d, 14 d, and 28 d, the ultimate load of the SHCC-0%RBP is respectively 5.18 kN, 6.22 kN, and 7.70 kN, and the results are 4.9 kN, 6.28 kN, and 6.94 kN for the SHCC-54%RBP. It is noticed that the ultimate displacement generally decreases with increasing curing days, and this is due to the appearance of embrittlement with aging [51]. Compared with the results in Figure 11a–d, the adverse effects of embrittlement on the ultimate displacement of the SHCC-0%RBP is higher than that of the SHCC with RBP; for example, there are only three peaks in the force-displacement curve of SHCC-0%, whereas there are six peaks in the force-displacement curve of SHCC-54% after 28d curing. This is because the hydration products in SHCC are decreased with increasing RBP replacements, and thus, the adverse impact of embrittlement caused by the increasing hydration reaction is reduced. 

**Figure 12:**
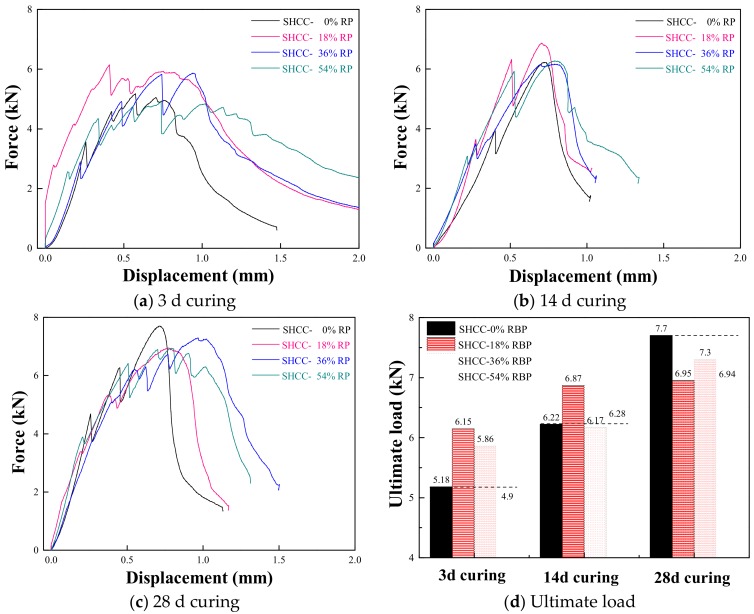
Force-displacement curves of SHCC with various RBP replacements. (**a**) 3 d curing; (**b**) 14 d curing; (**c**) 28 d curing; (**d**) Ultimate load.

To investigate the effects of RBP replacements on the flexural performance of SHCC, Figure 12 shows the force-displacement of SHCC containing various RBP contents. When the curing duration are 3 d, the ultimate load of SHCC-18%RBP and SHCC-36%RBP is higher than the SHCC-0%; however, when the RBP replacement ratios are 54%, the ultimate load of SHCC-54%RBP is lower than that of the SHCC-0%. This is because there is enough hydrated substance in the cementitious materials at the early age of curing; in this case, the appropriate content of RBP with high fineness and micro-hardness is beneficial to the mechanical properties of prepared SHCC. However, the addition of high-volume RBP results in a significant reduction in the hydration products, and thus the mechanical properties of SHCC are decreased. When the curing duration are 28 d, the ultimate load of SHCC with RBP is lower than the SHCC without RBP; this mainly due to that the hydrated substance decreases with the addition of RBP at the late period of curing, and the mechanical properties of prepared SHCC are decreased. Compared with the results in Figure 9 and Figure 12, the adverse effect of RBP replacement on the ultimate load of SHCC is much lower than that of the mortar after undergoing the same curing days; for example when the curing duration are 28 d, the ultimate load of the mortar with 18–54% RBP is 8.5–29.3% lower than that of the mortar without RBP, whereas the ultimate load of SHCC with 18–54%RBP is 5.2–9.9% lower than that of SHCC without RBP. Therefore, using RBP as SCM to prepare SHCC is more feasible than using RBP to prepare mortar and concrete. 

**Figure 13:**
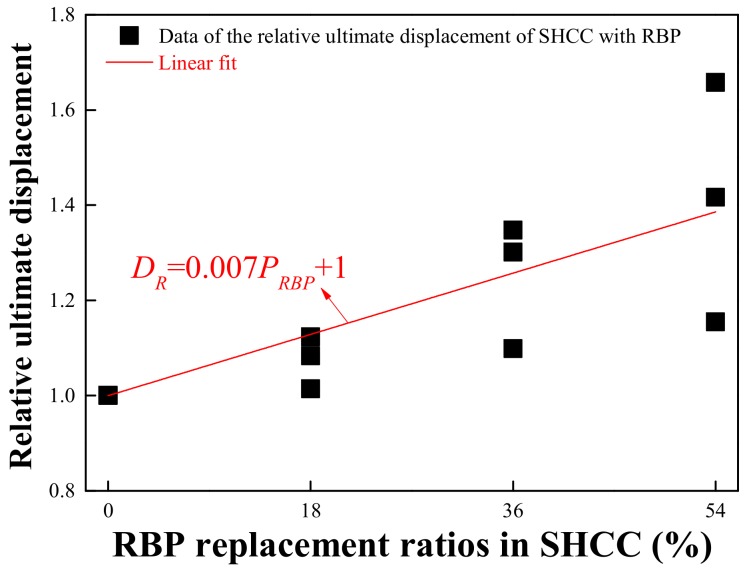
Relationship between the ultimate displacement of SHCC and and RBP replacements.

Considering Figure 12, the ultimate displacement of SHCC increases with increasing RBP replacements after undergoing the same curing days; for example, the ultimate displacement of SHCC-18%, SHCC-36%, and SHCC-54% is 12.3%, 30.1%, and 65.8% higher respectively, than that of the SHCC-0%RBP after 3 d curing, and the results are 8.3%, 34.7%, and 41.7% after 28 d curing. This may be because the high micro-hardness of SiO_2_, contained in RBP, with irregular and amorphous structure improves the bridging stress between the PVA fiber and cementitious materials, and thus the strain hardening properties of SHCC are improved with the addition of RBP. Furthermore, Figure 13 shows the relationship between the relative ultimate displacement of SHCC and the RBP replacements, and a linear relationship can be observed between them. The specific equation is also described in Figure 13, where the *D_R_* represents the relative value of the ultimate displacement of SHCC with RBP; *R_RBP_* is the RBP replacement ratios, in %. 

When using RBP to partially replace the cement by weight in SHCC, the results in this section highlight that the incorporation of RBP improves the strain hardening properties of prepared SHCC. In addition, compared with the flexural performance of SHCC without RBP, there is no obvious decrease in the ultimate load when the RBP replacement ratios are 54% in SHCC. Thus, using RBP as SCM to prepare SHCC is feasible, and the high-volume replacements of RBP derived from CDW decrease the demand of cement and provide an effective way of recycling the waste bricks in CDW.

### 3.4. Flexural Strength of SHCC when Using RBP to Replace Fly Ash by Weight

**Figure 14:**
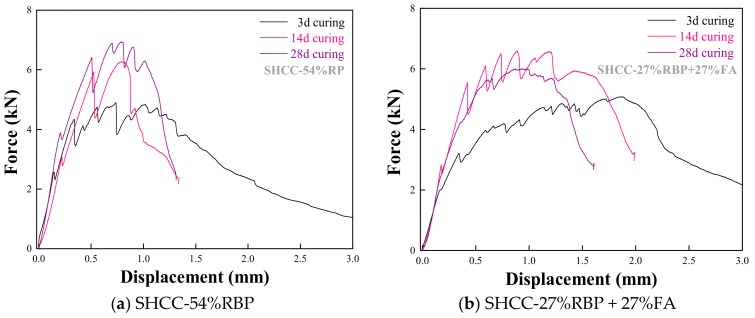
Force-displacement curves of SHCC with RBP and FA after various curing days. (**a**) SHCC-54%RBP; (**b**) SHCC-27%RBP + 27%FA.

**Figure 15:**
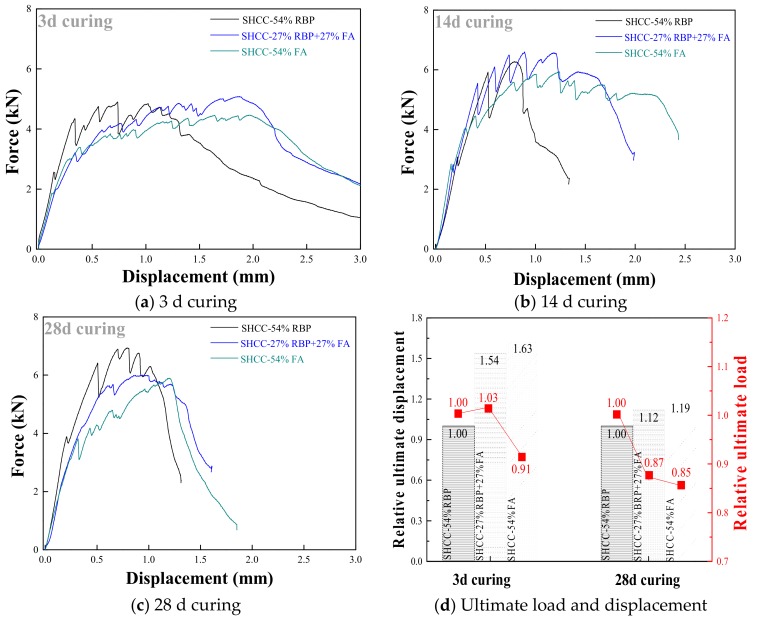
Force-displacement curves of SHCC when replacing FA with various RBP replacements. (**a**) 3 d curing; (**b**) 14 d curing; (**c**) 28 d curing; (**d**) Ultimate load and displacement.

This section investigates the flexural performance of SHCC, when using RBP to replace FA by weight in SHCC. Figure 14 shows the force-displacement curves of SHCC with FA and RBP after various curing days, and the results show that the ultimate load increases and the ultimate displacement decreases with the increase of curing days. Figure 15 further shows the force-displacement curves of SHCC when replacing FA with various contents of RBP. The ultimate load of SHCC with RBP is higher than that of SHCC with FA; for example, the ultimate load of SHCC-54%RBP after 3 d, 14 d and 28 d curing were 9.8%, 5.8%, and 17.6% higher, respectively, than that of SHCC-54%FA. This is because the content of SiO_2_ micro-particle in RBP is higher than that in FA (as shown in Figure 2b), and the higher fineness SiO_2_ with high micro-hardness is beneficial to the mechanical properties of SHCC. However, the ultimate displacement of SHCC with RBP is lower than that of SHCC with FA after undergoing the same curing days. In particular, the difference between the ultimate displacement of SHCC with FA and RBP is decreased with increasing curing days. For example, the ultimate displacement of SHCC-27%RBP + 27%FA and SHCC-54%RBP were 5.6% and 38.6% lower than that of SHCC-54%FA after 3 d curing, and the results were 6.6% and 16.4% after 28 d curing. 

The mechanism of the improvement in the strain hardening characteristic is different for the SHCC with FA and RBP. For the SHCC with FA, the microsphere structure of FA well disperses the PVA fibers in SHCC, and the addition of FA improves the ductility of cementitous materials. Thus, the strain hardening characteristic of SHCC increases with the incorporation of FA [52,53]. However, for the SHCC with RBP, the improvement in the strain hardening characteristic is mainly contributed to its amorphous structure and high micro-hardness; in this case, the bridging stress between the fiber and cementitious materials is improved, and the ultimate stress and strain are both improved. Therefore, the ultimate load of SHCC with RBP is higher than that with FA, whereas a contrary phenomenon is observed for the ultimate displacement. When using RBP to completely replace the FA in SHCC, the ultimate load and displacement of SHCC-54%RBP are 17.6% higher and 16.4% lower than that of SHCC-54%FA. Therefore, the RBP was an appropriate SCM to prepare SHCC; especially for the SHCC may suffer a high ultimate load, the applicability of RBP is higher than that of FA in SHCC preparation.

Besides, the economic and environmental benefits of CBP preparation are higher than that of cement preparation [54]. The previous findings report that the energy consumption, CO_2_ emission and cost of cement production are respectively 5.5 MJ/kg, 0.930 kg/kg, and 500 RMB/ton, and the results are 0.1–1.2 MJ/k, 0.028–0.333 kg/kg, and 30–330 RMB/ton for the preparation of RBP with various particle sizes [55]. Therefore, the CBP is an eco-friendly recycled material, and the utilization of CBP in SHCC preparation has excellent economic and environmental benefits. 

## 4. Conclusions

Using RBP as SCM to prepare SHCC provides a new way of recycling the CDW and decreases the dosage of cement in SHCC preparation. The micro and mechanical properties of SHCC with RBP were determined in this paper. Based on the results and discussions, the following conclusions can be obtained.

(1) The RBP has higher silicon content and lower calcium content than the cement and FA. The addition of RBP increases the content of SiO_2_ in the cementitious materials, however, the hydrated compound and hydration products are decreased with the addition of RBP. The high fineness RBP has the typical characteristic of SCM, and it is feasible to use RBP as SCM in the preparation of cementitious composites.
(2) The incorporation of RBP decreases the compressive strength and flexural strength of the prepared mortar, and a linear relationship can be observed between them. However, the adverse effect of RBP on the mechanical properties is decreased with increasing PVA fiber content; moreover, the ultimate load and displacement both increases with the increase of PVA fiber content, and an excellent strain hardening characteristic appears when the content of PVA fiber is 26 g.
(3) Using RBP as SCM to partially replace the cement in SHCC: The ultimate load increases and the ultimate displacement decreases with increasing curing days. There is no obvious reduction in the ultimate load of SHCC with the addition of RBP. The ultimate displacement of SHCC increases with increasing RBP replacements, which indicates that the incorporation of RBP improves the ductility of SHCC. The ultimate displacement of SHCC-54%RBP is 41.7–65.8% higher than that of SHCC-0% after various curing days.
(4) When using RBP to replace FA in SHCC: Although replacing FA by the same weight of RBP decreases the ultimate displacement of SHCC, the ultimate load is increased. The ultimate load and displacement of SHCC-54%RBP are 17.6% higher and 16.4% lower than that of SHCC-54%FA. Under some specific conditions (such as those with a requirement of high strength), the SHCC with RBP is more suitable applied in the construction than the SHCC with FA.
(5) Although this paper proved the feasibility of using RBP to prepare SHCC, there still exist some shortcomings which should be studied in the future. For example, the particle size and types of recycled powder, derived from CDW, may significantly impact the micro and mechanical properties of prepared SHCC. Moreover, the uniaxial tensile test should be employed in the determination of properties.

## Figures and Tables

**Table 1:** Mix proportion of the mortar and SHCC with RBP (g).

Sample	Cement	RBP	FA (Class I)	Water	Sand	PVA Fiber
Mortar-0%RBP	1200	0	0	395	550	0
Mortar-18%RBP	984	216				
Mortar-36%RBP	768	432				
Mortar-54%RBP	550	650				
Mortar-0 F	550	650	0	395	550	0
Mortar-6.5 F						6.5
Mortar-13 F						13
Mortar-26 F						26
SHCC-0%RBP	1200	0	0	395	550	26
SHCC-18%RBP	984	216				
SHCC-36%RBP	768	432				
SHCC-54%RBP	550	650				
SHCC-27%RBP + 27%FA	550	325	325	395	550	26
SHCC-54%FA	550	0	650			
